# Comparison of Survivin Determination by Surface-Enhanced Fluorescence and Raman Spectroscopy on Nanostructured Silver Substrates

**DOI:** 10.3390/bios14100479

**Published:** 2024-10-06

**Authors:** Georgia Geka, Anastasia Kanioura, Ioannis Kochylas, Vlassis Likodimos, Spiros Gardelis, Anastasios Dimitriou, Nikolaos Papanikolaou, Anastasios Economou, Sotirios Kakabakos, Panagiota Petrou

**Affiliations:** 1 Immunoassays/Immunosensors Laboratory Institute of Nuclear & Radiological Sciences & Technology, Energy & Safety, NCSR “Demokritos”, 15341 Aghia Paraskevi, Greece; g.gkeka@rrp.demokritos.gr (G.G.); skakab@rrp.demokritos.gr (S.K.); 2 Department of Chemistry, National and Kapodistrian University of Athens, University Campus, 15771 Athens, Greece; aeconomo@chem.uoa.gr; 3 Section of Condensed Matter Physics, Department of Physics, National and Kapodistrian University of Athens, University Campus, 15784 Athens, Greece; ikochyla@phys.uoa.gr (I.K.); vlikodimos@phys.uoa.gr (V.L.); sgardelis@phys.uoa.gr (S.G.); 4 Institute of Nanoscience & Nanotechnology, NCSR “Demokritos”, 15341 Aghia Paraskevi, Greece; a.dimitriou@inn.demokritos.gr (A.D.); n.papanikolaou@inn.demokritos.gr (N.P.)

**Keywords:** survivin, immunochemical detection, surface-enhanced photoluminescence, surface-enhanced Raman spectroscopy, optical biosensor, cancer marker

## Abstract

Survivin belongs to a family of proteins that promote cellular proliferation and inhibit cellular apoptosis. Its overexpression in various cancer types has led to its recognition as an important marker for cancer diagnosis and treatment. In this work, we compare two approaches for the immunochemical detection of survivin through surface-enhanced fluorescence or Raman spectroscopy using surfaces with nanowires decorated with silver nanoparticles in the form of dendrites or aggregates as immunoassays substrates. In both substrates, a two-step non-competitive immunoassay was developed using a pair of specific monoclonal antibodies, one for detection and the other for capture. The detection antibody was biotinylated and combined with streptavidin labeled with rhodamine for the detection of surface-enhanced fluorescence, while, for the detection via Raman spectroscopy, streptavidin labeled with peroxidase was used and the signal was obtained after the application of 3,3′,5,5′-tetramethylbenzidine (TMB) precipitating substrate. It was found that the substrate with the silver dendrites provided higher fluorescence signal intensity compared to the substrate with the silver aggregates, while the opposite was observed for the Raman signal. Thus, the best substrate was used for each detection method. A detection limit of 12.5 pg/mL was achieved with both detection approaches along with a linear dynamic range up to 500 pg/mL, enabling survivin determination in human serum samples from both healthy and ovarian cancer patients for cancer diagnosis and monitoring purposes.

## 1. Introduction

Survivin is a small protein (MW 16.5 kDa) that plays a key role in the process of programmed cell death (apoptosis) and the regulation of cell division [1,2,3,4]. It is encoded by the BIRC5 gene located on chromosome 17 (17q25) and therefore is usually referred to as the BIRC5 protein. Survivin overexpression has been correlated with poor prognoses of many malignancies, such as lung, colon, bladder, prostate, and ovarian cancers [1,2,3]. Moreover, survivin has been identified as a target for cancer therapeutics [5,6], and several approaches to this direction have been explored, such as the implementation of inhibitors against survivin homodimerization or interaction with other proteins, inhibitors of survivin expression, and immunotherapy [6].

The normal-range cut-off value for survivin in human serum is set between 110 and 120 pg/mL [7,8]. In addition to serum, the determination of the survivin levels in urine samples has been implemented, especially in cases of bladder cancer [9,10,11,12,13], as a non-invasive method for early diagnosis and treatment monitoring, employing enzyme immunoassay kits (ELISAs) developed for measurements both in serum and urine. Survivin has also been used as a marker for canine cancer diagnosis by implementing immunochemical methods [14]. The greatest advantage of ELISAs for the determination of the disease markers in human biological samples is that the specificity of the antibody–antigen binding reaction is fully exploited by the immunosensors, which, in addition to selectivity, offer ease of use, lower analysis costs, automation, portability, and reusability [15]. In immunosensors, contrary to immunoassays, the substrate plays the major role in the overall assay performance and especially in detection sensitivity. This is particularly true for surface-enhanced Raman spectroscopy (SERS) [15,16,17,18] and surface-enhanced fluorescence (SEF) [19,20,21,22,23], which rely on phenomena of optical signal amplification that take place on nanostructured metallic surfaces, which can have various shapes and sizes but are mostly composed of either Au or Ag [24,25,26].

The mechanism behind SERS and SEF on a nanostructured metallic substrate is surface plasmon resonance, which occurs via the collective oscillations of the metal conductive electrons upon irradiation with light of appropriate wavelength [17,18]. These oscillations generate a strong electromagnetic field at the metal–dielectric interface that, in the case of continuous metal surfaces, propagates as a longitudinal wave and is known as surface plasmon resonance (SPR), whereas, in non-continuous (i.e., nanostructured) substrates, it is highly localized on the surface structures and is referred to as localized SPR (LSPR). LSPR is responsible for the enhancement of the Raman or photoluminescence signals of molecules directly attached or immunoadsorbed on the metal nanostructures.

Both detection approaches have been used to detect molecules in biological or other samples implementing specific recognition molecules, mainly antibodies [15,16,21,22]. The determination of cancer markers is one of the application areas where detection either by SERS or SEF onto nanostructured metallic surfaces has been explored [15,27,28,29,30,31,32]. The motivation is the achievement of higher detection sensitivity that in many cases is crucial for early diagnosis and monitoring of therapy [27,28,29,30,31,32], as well as the fact that both detection principles could be adapted for multiplexed determination of more than one cancer biomarker simultaneously [31] and/or integrated on microfluidic devices for rapid determinations at the point of care.

**Figure 1:**
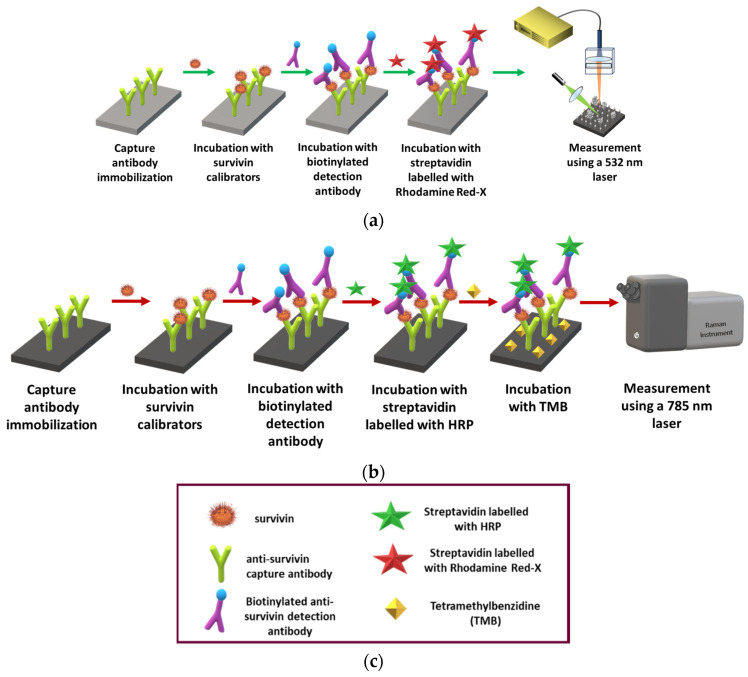
Schematic illustration of the immunoassay for survivin determination with SEF (**a**) or SERS (**b**) and of the reagents used in both assays (**c**).

Thus, in this work, the immunochemical detection of survivin through surface-enhanced fluorescence and Raman spectroscopy is presented using nanostructured silicon substrates with two different types of silver particles on top, namely dendrites and aggregates. Both types of substrates were prepared using metal-assisted chemical etching (MACE) that employs treatment of silicon wafers with a mixture of hydrofluoric acid and aqueous silver nitrate solution, during which two different phenomena take place, silver nucleation, leading to the creation of silver nanoparticles, and silicon etching, leading to silicon nanowires [33,34,35,36]. The result is a substrate of silicon nanowires with silver dendrites on top [33,34]. The removal of the formed silver structures through acidic treatment and re-immersion in the hydrofluoric acid/aqueous silver nitrate solution give rise to the formation of new silver nanoparticles on top of the silicon nanowires. The silver nanoparticles created in this second immersion in hydrofluoric acid/aqueous silver nitrate solution have the form of nanoparticle aggregates. Both types of substrates have been evaluated for the detection of biomolecules via SERS or SEF either after their direct adsorption onto the surface or following immunochemical approaches. In the present work, the nanostructured silver/silicon (Ag/Si) substrates with dendrites or aggregates were used as substrates to develop and compare the survivin assay based on SEF or SERS. Both assays were realized using a pair of rabbit monoclonal antibodies. The detection antibody was biotinylated to enable detection by fluorescence after reaction with fluorescently labeled streptavidin. On the other hand, for the detection through Raman spectroscopy, two different labeling approaches were investigated: streptavidin was conjugated to gold nanoparticles modified with a Raman reporter molecule (4-mercaptobenzoic acid), or a peroxidase-labeled streptavidin was used in combination with a precipitating peroxidase substrate that provided a characteristic Raman spectrum. The main steps of the two immunoassay procedures and the respective signal detection are schematically depicted in Figure 1. Following the optimization of all the immunoassay parameters, the analytical performance of the two detection approaches, i.e., fluorescence versus Raman, was compared.

## 2. Materials and Methods

### 2.1. Materials

P-type (100)-oriented monocrystalline Si wafers were from Silicon Materials (Kaufering, German). Silver nitrate (AgNO_3_; ≥99.0%), 3-mercaptopropyl-trimethoxysilane (3-MPTMS; 95%), streptavidin-peroxidase polymer (streptavidin-HRP), 4-mercaptobenzoic acid (4-MBA), gold nanoparticles (20 nm diameter, OD 1, stabilized suspension in 0.1 mM PBS, reactant free), and TMB Enhanced One Component HRP Membrane Substrate (TMB precipitating substrate) were from Sigma-Aldrich (Darmstadt, Germany). Hydrofluoric acid (HF; 50% in water) was from Technic Inc. (Saint-Denis, France). Recombinant rabbit monoclonal antibodies against human survivin (codes ab242675 and ab242920 as capture and detection antibodies, respectively) as well as recombinant human survivin were purchased from Abcam (Cambridge, UK). Streptavidin conjugated with Rhodamine Red-X and EZ-Link™ Sulfo-NHS-LC-Biotin (sulfo-NHS-biotin) were from ThermoFischer Scientific (Waltham, MA, USA). Bovine serum albumin (BSA) was from Acros Organics (Geel, Belgium). Lipid-stripped human serum was from Scantibodies Laboratory, Inc. (Santee, CA, USA). Twenty-four-well culture plates were from Orange Scientifique NV (Braine-l’Alleud, Belgium). All other chemicals were of analytical grade from Merck (Darmstadt, Germany). The water used throughout the study was distilled.

### 2.2. Preparation of Nanostructured Ag/Si Substrates and Modification with 3-Mercaptopropyl-trimethoxysilane (3-MPTMS)

The nanostructured Ag/Si substrates were fabricated by metal-assisted chemical etching (MACE) that has been optimized for SEF and SERS measurements as described in previous works [33,34]. In brief, for the preparation of nanostructured Ag/Si substrates for SEF measurements, the Si wafers were immersed for 3.5 min in a 0.02 M AgNO_3_, 4.8 M HF solution. After that, they were washed extensively with deionized water and left to dry. The preparation of nanostructured Ag/Si substrates for SERS measurements included immersion in the same solution for 6 min, followed by immersion for 4 min in a 50% *v/v* aqueous HNO_3_ solution and again immersion in the AgNO_3_/HF solution for 7 s. Then, the substrates were washed with deionized water and left to dry. A scanning electron microscope (JSM-7401F SEM; JEOL Europe bv, Zaventem, Belgium) working at 30 kV was employed to acquire images of the nanostructured Ag/Si surfaces. The process followed for the preparation of the nanostructured Ag/Si substrates is schematically depicted in Figure S1.

Modification of nanostructured Ag/Si substrates with 3-MPTMS was achieved by immersion for 2 h in a 4% *v/v* 3-MPTMS solution in absolute ethanol followed by washing in ethanol and drying under a nitrogen stream.

### 2.3. Biotinylation of Anti-Survivin Detection Antibody

The rabbit monoclonal antibody against survivin with clone code ab242920 was biotinylated in order to be used as detection antibody in both SEF and SERS immunoassays. At first, the antibody solution was dialyzed against 0.25 M carbonate buffer, pH 9.2, 9 g/L NaCl, and then a 100 μg/mL solution of sulfo-NHS-biotin in dimethyl sulfoxide was added for the weight ratio of sulfo-NHS-LC-biotin to antibody in the reaction mixture to be 2:1. The reaction mixture was left for 2 h at room temperature (RT), prior to dialysis against a 0.1 M NaHCO_3_ solution, pH 8.5, 9 g/L NaCl, 0.5 g/L NaN_3_. After that, the biotinylated anti-survivin antibody solution was collected and kept at 4 °C.

### 2.4. Preparation of Raman Label

Gold nanoparticle labeling with 4-MBA and streptavidin was performed following a slight modification of a published protocol [37]. Briefly, 10 μL of 1.0 mM 4-MBA solution in absolute ethanol was added to 1.0 mL of gold nanoparticle (AuNP) solution and the mixture was incubated overnight at RT. After centrifugation at 8000× *g* for 20 min, the 4-MBA-AuNPs were dispersed in 0.5 mL of 10 mM MES buffer, pH 5.5, and mixed with 10 μL of 0.1 M EDC and 20 μL of 0.1 M sulfo-NHS solutions in the same buffer. After incubation for 30 min, the mixture was centrifuged twice at 10,000× *g* for 20 min and resuspended to 0.5 mL of 10 mM PBS buffer, pH 7.4. Then, 20 μL of a 5.0 mg/mL streptavidin solution were added to 4-MBA-AuNPs solution and the mixture was incubated for 2 h at 4 °C prior to addition of 100 μL of a 50 mg/mL BSA solution and further incubation overnight at 4 °C. After that, the 4-MBA-AuNPs-streptavidin conjugate solution was centrifuged and resuspended twice in 0.5 mL of 10 mM PBS, pH 7.4, containing 10 mg/mL BSA and kept at 4 °C until use.

### 2.5. Survivin Immunoassay on Nanostructured Ag/Si Substrates

For the immunoassay, 0.5 × 0.5 cm^2^ dies of nanostructured Ag/Si substrates were placed onto 24-well polystyrene plates. The dies were incubated with 10 μL of a 100 μg/mL anti-survivin capture mAb solution in 50 mM phosphate buffer, pH 7.4, overnight at 4 °C, and then washed twice with 300 μL of a 10 mM Tris-HCl buffer, pH 8.25, 9 g/L NaCl (washing solution). After that, 300 μL of a 10 mg/mL BSA in 0.1 M NaHCO_3_, pH 8.5 (blocking solution), were added per well and incubated for 2 h at RT. After washing as previously, 200 μL of survivin calibrators in 50 mM Tris-HCl, pH 7.8, 10 g/L BSA, 9 g/L NaCl, 0.5 g/L NaN_3_ (assay buffer) were added in each well and the dies were incubated for 1 h at RT under gentle shaking. Then, after washing, 200 μL of a 1.25 μg/mL solution of biotinylated anti-survivin detection antibody in assay buffer were added per well and incubated for 1 h at RT under shaking. The substrates were then washed with washing solution containing 0.5 mL/L Tween 20 (3 × 300 μL) and, depending on the detection principle, SEF or SERS were incubated with 200 μL of a 5 μg/mL streptavidin-Rhodamine Red-X solution or a 25 ng/mL streptavidin-HRP solution in 50 mM PBS, pH 6.5, 10 g/L BSA for 30 min at RT under shaking. After that, on the substrates incubated with streptavidin-Rhodamine Red-X, measurements of SEF signal intensity were performed, whereas those reacted with streptavidin-HRP were incubated with 200 μL of the TMB precipitating solution for 5 min prior to washing with distilled water (3 × 300 μL) and drying.

### 2.6. SEF Measurements

SEF measurements were performed using an in-house-developed set-up involving a green diode laser at 532 nm for sample illumination so as to match as possible the Rhodamine Red-X excitation maxima at 533 and 571 nm. Surface illumination was performed at a 45° angle through a focusing lens, while the light emitted from the sample was collected by an optical fiber through a long pass filter at 550 nm (Ocean Insight; Duiven, Netherlands) (Figure 1a). The illuminated area diameter was approximately 2 mm and the laser power on the sample 2 mW. To prepare the survivin calibration curve, 10 spectra were collected from each one of 3 dies reacted with the different calibrators and the mean maximum value of the SEF signal corresponding to each calibrator was calculated. By subtracting from the mean SEF signal of each calibrator, the zero calibrator value, i.e., the signal received in absence of the analyte, and the net signals were obtained and plotted against survivin concentration in the calibrators to receive the calibration curve.

### 2.7. Raman Measurements

The SERS measurements were conducted using an inVia Reflex microscope (Renishaw, UK) that had a solid-state laser operating at 785 nm so as to minimize interference on the Raman lines from the substrate autofluorescence (Figure 1b). The laser was focused on the samples with an x20 (ΝA = 0.4) objective lens on a Leica DMLC microscope, resulting in a spot size of approximately 1 μm. The laser’s power density was set to 0.06 mW/μm^2^ and each measurement lasted for 10 s. A total of ten spectra were acquired for each die, and three replicates were used for all cases unless stated otherwise. The average of the values received from the repetitive measurement for the peak at 1607 cm^−1^ was utilized for the fabrication of the survivin calibration curve after the removal of the corresponding zero calibrator signal.

## 3. Results

### 3.1. Nanostructured Ag/Si Substrates

**Figure 2:**
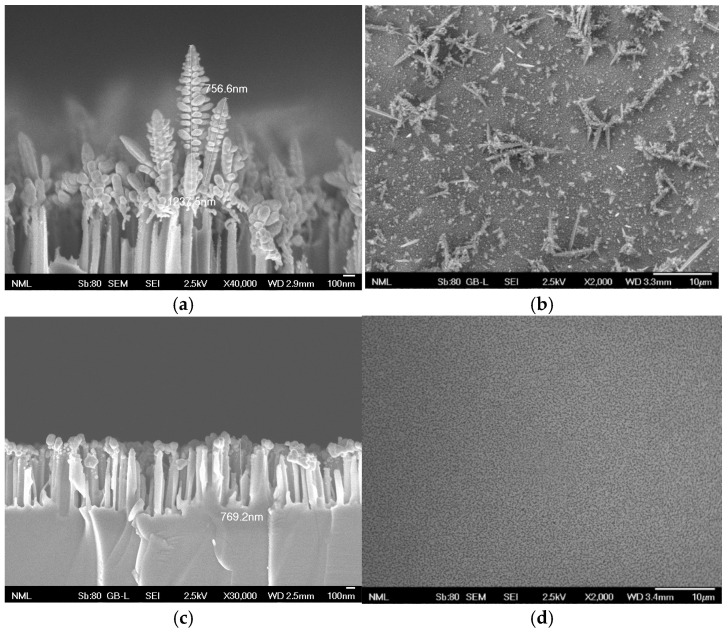
Characteristic cross and top view SEM images of Si nanowires with height of approximately 250 nm decorated with 800 nm-long Ag dendrites (**a**,**b**) or Si nanowires with height of approximately 500 nm decorated with approximately 150 nm-long Ag aggregates (**c**,**d**). The cross-view images (**a**,**c**) have been received with a higher magnification to show in detail the Ag nanoparticles’ structure whereas the top view images (**b**,**d**) with a lower magnification to demonstrate the distribution of these structures across the substrate surface.

The nanostructured Ag/Si substrates used in this study were prepared following the MACE procedure, the basic step of which is the treatment of the Si wafers in an aqueous mixture of AgNO_3_ and HF [33,34]. During this step, etching of the Si takes place, leading to Si nanowire formation with the simultaneous development of Ag nanoparticles at the top of the Si nanowires. The Ag nanoparticles developed are received in the form of dendrites, as shown in the SEM images provided in Figure 2. The length of the Si nanowires did not exceed 250 nm, while the height of the majority of the dendritic Ag structures was around 1 μm (Figure 2a). However, as shown in the top view SEM image of Figure 2b, Ag agglomerates with heights up to 10 μm were also created, leading to a highly inhomogeneous surface. To create Ag nanoparticles of different structures, the immersion in the AgNO_3_/HF solution was increased to 6 min, leading to a mean height of the Si nanowires ranging from 400 to 500 nm. Then, the Ag dendrites formed on the tips of these Si nanowires were dissolved, and Ag nanoparticles in the form of aggregates with diameters of about 150 nm (Figure 2c) were created by short immersion in the AgNO_3_/HF solution. In this case, the surface was homogeneously covered with Ag nanoparticles, as indicated by the top view SEM image in Figure 2d. However, a top view image of this surface received in higher magnification reveals the fine structure of the Ag nanoparticles created on the etched silicon substrate (Figure S2).

Despite their inhomogeneity, the surfaces with the Ag dendrites provide higher enhancement of the fluorescence signal as compared to the surfaces with the Ag aggregates, while the latter perform better as substrates for SERS. As shown in Figure S3a, for the 100 pg/mL survivin calibrator, the fluorescence signal intensity at 590 nm (peak of fluorescence spectrum) received from the substrates with the dendrites was approximately 35% higher than that received from the substrates with the aggregates. On the other hand, the substrates with the aggregates provided approximately 40% higher Raman signals (taking into account the peak at 1607 cm^−1^) compared to the values obtained from the substrates with the dendrites (Figure S3b). This finding is consistent with our previous findings regarding the enhancement of the fluorescence or Raman signals from nanostructured Ag/Si substrates [36], and it can be ascribed to the different mechanisms behind the two phenomena. Thus, the SEF signal is maximized at a certain distance from the substrate due to the minimization of the signal quenching that occurs when the fluorophores are located close to the metal structures [33,34]. On the other hand, the SERS signal is the result of strong localized electric fields generated on the nanometer scale (hot spots) when the light illuminating the surface leads to the resonance of metal nanostructure surface plasmons. The enhancement increases as the distance between the hot spots and the hot spots and molecules decreases [33,34]. The SERS enhancement factor of the surfaces used in the current study was calculated as described in the Supplementary Materials (Figure S4) following a published method [37] and was found to be in the range of 10^8^ (Table S1). Therefore, nanostructured substrates with Si nanowires with Ag dendrites and aggregates were used for the development of the immunochemical methods for the determination of survivin by SEF and SERS, respectively.

### 3.2. Selection of Survivin Immunoassay Configuration

**Figure 3:**
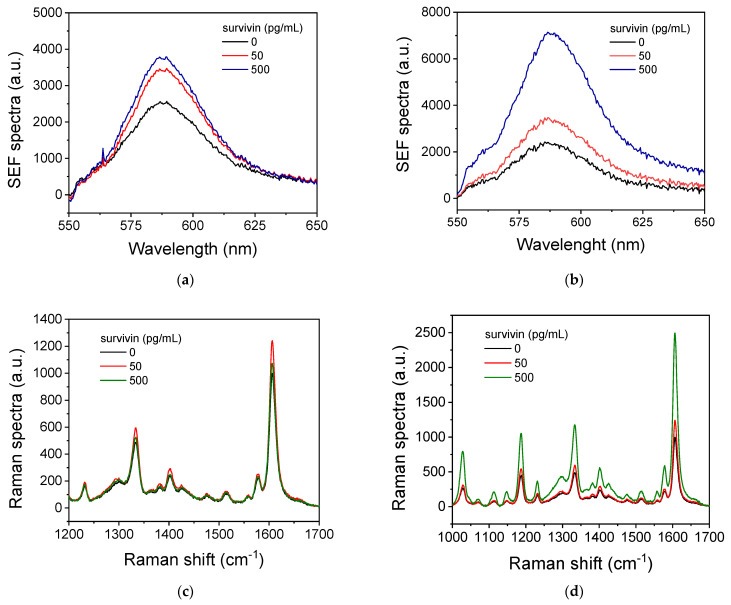
(**a**,**b**) SEF spectra obtained from nanostructured Ag/Si substrates with Ag dendrites following a one-step (**a**) or a two-step survivin immunoassay (**b**) for the zero calibrator (black line) and calibrators containing 50 (red line) and 500 pg/mL (blue line). (**c**,**d**) SERS spectra obtained from nanostructured Ag/Si substrates with Ag aggregates following a one-step (**c**) or a two-step survivin immunoassay (**d**) for the zero calibrator (black line) and calibrators containing 50 (red line) and 500 pg/mL (green line).

The immunochemical determination of survivin by SEF and SERS was based on a non-competitive assay using a pair of two rabbit monoclonal antibodies against survivin. At first, the non-competitive immunoassay format, i.e., if the immunoassay could be performed in one or two steps, was determined. The one-step immunoassay includes incubation of the immobilized antibody (capture antibody) on the substrate with a mixture of the calibrator/sample with the biotinylated detection antibody. On the other hand, in the two-step immunoassay, the capture antibody reacts first with the analyte in the calibrator/sample and then, in a second step, the detection antibody is added and reacts with the analyte molecules that have been bound on the capture antibody during the first step. In Figure 3, the SEF (Figure 3a,b) and SERS spectra (Figure 3c,d) obtained from nanostructured Ag/Si substrates with Ag dendrites and aggregates, respectively, for survivin calibrators with concentrations of 0, 50, and 500 pg/mL following either the one-assay (Figure 3a,c) or two-assay (Figure 3b,d) format are presented. In all the cases, the capture antibody was used at a concentration of 100 μg/mL and the detection antibody at 2.5 μg/mL. The one-step immunoassay lasted for 1 h, whereas, in the two-step immunoassay, the duration of each step was 1 h. As shown, the one-step and the two-step immunoassays provided similar values for the zero calibrator and the calibrator containing 50 pg/mL survivin for detection by either SEF or SERS. However, for the higher-concentration calibrator (500 pg/mL), the one-step immunoassay provided a significantly lower signal compared to that received by the two-step immunoassay. Additionally, in the case of SERS, the signal received for the 500 pg/mL calibrator was lower than the signal corresponding to the 50 pg/mL calibrator. This phenomenon is known as the high-dose hook effect and is attributed either to a limited amount of the detection antibody with respect to the analyte or to some overlapping of the epitopes recognized by the two antibodies in the analyte molecule. This overlapping leads to competition between the two antibodies for binding to the analyte rather than a synergistic effect when a one-step non-competitive immunoassay format is followed. To ensure that the high-dose hook effect was not due to a limited amount of the detection antibody, concentrations ranging from 2.5 to 10 μg/mL were tested. This increase in the detection antibody concentration helped to increase the signal for the 500 pg/mL calibrator in the one-step immunoassay compared to that of the 50 pg/mL calibrator but did not reach the signal obtained for the 500 ng/mL calibrator by the two-step assay format. Thus, the two-step assay was selected for further experimentation.

### 3.3. Survivin SEF Immunoassay Optimization

The survivin immunoassay was separately optimized onto nanostructured Ag/Si substrates with Ag dendrites or Ag nanoparticles with respect to parameters such as the capture antibody concentration used for the coating of the dies, the concentration of the biotinylated detection antibody, the duration of the two immunoassay steps, and the incubation time with the labeled streptavidin used in each detection approach. Some other parameters, however, including the composition of all the solutions used, were common for both detection approaches.

Regarding the composition of the buffer used for the preparation of the capture antibody solution, three different buffers were tested: phosphate buffer 50 mM, pH 7.4; carbonate buffer 50 mM, pH 9.2; and Tris-HCl buffer 10 mM, pH 8.25, 0.1 M NaCl. The SEF signals received for the zero calibrator and a calibrator containing 50 pg/mL survivin are presented in Figure S5. As shown, the buffer that provided the lowest non-specific signal and the highest specific signal was the phosphate buffer 50 mM, pH 7.4. The assay buffer was also selected between 50 mM Tris-HCl, pH 7.8, 10 g/L BSA, 9 g/L NaCl, 0.5 g/L NaN_3_, and 50 mM PBS, pH 7.4, 10 g/L, BSA, 0.5 g/L NaN_3_. As presented in Figure S6, the Tris-HCl buffer (50 mM Tris-HCl, pH 7.8, 10 g/L BSA, 9 g/L NaCl, 0.5 g/L NaN_3_) provided higher values compared to the PBS buffer for the whole range of survivin concentrations tested. Thus, it is expected that it would also provide high detection sensitivity and was therefore selected as the assay buffer.

**Figure 4:**
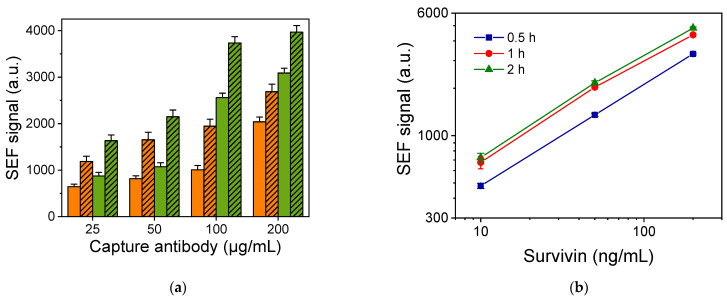
(**a**) SEF signal values corresponding to zero calibrator (plain orange and green columns) and a calibrator containing 50 pg/mL of survivin (striped orange and green columns) obtained using the detection antibody at concentrations of 1.25 (orange columns) and 2.5 μg/mL (green columns). Each point is the mean of three samples ± SD. (**b**) Survivin calibration curves obtained from nanostructured Ag/Si substrates with Ag dendrites for duration of each immunoassay step equal to 0.5 (squares), 1 (circles), or 2 h (triangles). Each point is the mean of three samples ± SD.

The next parameters to be optimized were the concentrations of the capture and detection antibodies. In Figure 4a, the signals received for the zero calibrator (non-specific signal) and a calibrator containing 50 pg/mL of survivin using different combinations of capture and detection antibody concentrations are provided. As shown, the increase in the concentration of either the capture or the detection antibody increased the signals corresponding to both the zero and the 50 pg/mL calibrator. However, the net SEF signal for the 50 pg/mL calibrator (SEF signal of 50 pg/mL calibrator/SEF signal of zero calibrator) remained stable for capture antibody concentrations equal to or higher than 100 μg/mL and detection antibody concentrations equal to or higher than 1.25 μg/mL. Thus, this combination of capture and detection antibody concentrations was selected for the final immunoassay protocol. In a similar way, the concentration of the fluorescently labeled streptavidin was optimized. A 5 μg/mL streptavidin-Rhodamine Red-X concentration was selected since it provided the highest possible specific signal along with the lower non-specific binding signal.

The durations of the two immunoassay steps were also optimized. In Figure 4b, the SEF signals obtained for three survivin calibrators (10, 50, and 200 pg/mL), when the duration of each immunoassay step was 0.5, 1, or 2 h, respectively, are presented. As shown, an increase in each assay step duration from 0.5 to 1 h resulted in a 30 to 50% signal increase for all the calibrators. On the other hand, a further increase in each assay step duration to 2 h resulted in a marginal increase of 5 to 10% (depending on the calibrator) compared to a 1 h duration. Thus, in order to maximize the signal without unnecessarily increasing the whole assay duration, 1 h was selected for both assay steps. Regarding the last step of the assay, i.e., the incubation with streptavidin-Rhodamine X Red, a duration of 30 min was selected, for which maximum plateau values were obtained.

### 3.4. Optimization of Survivin SERS Immunoassay

**Figure 5:**
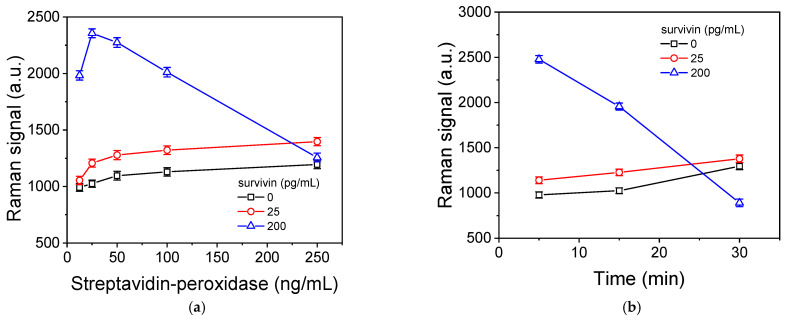
(**a**) Raman signals corresponding to peak at 1607 cm^−1^ obtained for the zero calibrator (squares) or calibrators containing 25 (circles) or 200 pg/mL (triangles) of survivin with respect to the streptavidin-peroxidase concentration. The incubation with the precipitating TMB substrate was 15 min. Each point is the mean of three samples ± SD. (**b**) Raman signals corresponding to peak at 1607 cm^−1^ obtained for the zero calibrator (squares) or calibrators containing 25 (circles) or 200 pg/mL (triangles) of survivin with respect to the duration of incubation with the precipitating TMB substrate. The streptavidin-peroxidase concentration was 25 ng/mL. Each point is the mean of three samples ± SD.

Most assay parameters, including the concentrations of the capture and detection antibodies, the composition of the buffers used, and the duration of the two immunoassay steps defined during the optimization of the survivin SEF immunoassay, were applied to the SERS substrates for the development of the respective immunoassays. The step that differs between the two detection approaches is the use of the streptavidin conjugate regarding the Au nanoparticles modified with 4-MBA (4-MBA-AuNPs-streptavidin) or streptavidin labeled with peroxidase in combination with the precipitating substrate (TMB) instead of the fluorescently labeled streptavidin [38,39]. As shown in Figure S7, the net Raman signal corresponding to a calibrator of 50 pg/mL is at least five times lower in the case of the 4-MBA-AuNPs-streptavidin compared to that obtained using streptavidin-peroxidase and the TMB precipitating substrate for signal acquisition. This was to a great extent expected since the use of the enzyme results in significant signal enhancement due to the continuous creation of the enzymatic reaction products over time. Thus, the latter method was selected for the development of the SERS assay, and the optimum streptavidin-peroxidase concentration and the incubation duration with the precipitating peroxidase substrate were determined. The concentration of streptavidin was determined taking into account the zero calibrator signal and the signals corresponding to low- (25 pg/mL) and high-concentration calibrators (200 pg/mL). It was found that the absolute zero and lower-concentration calibrator signals, as well as the respective net signal, increased and reached maximum plateau values for streptavidin-peroxidase concentrations equal to or higher than 100 ng/mL. On the contrary, the absolute and net signals corresponding to the high-concentration calibrators increased as the streptavidin-peroxidase concentration increased up to 50 ng/mL and then deceased as the streptavidin-peroxidase concentration further increased (Figure 5a). The same phenomenon was observed for a fixed streptavidin-peroxidase concentration of 25 ng/mL as the incubation time with the precipitating solution was increased from 5 to 30 min (Figure 5b).

To investigate the phenomenon of Raman signal drop due to prolonged incubation with the TMB precipitating substrate, SEM images of Ag/Si substrates were received from surfaces reacted with a 200 pg/mL survivin calibrator and then with a 25 ng/mL streptavidin-peroxidase solution prior to and after the application for 5 and 15 min of the precipitating TMB substrate. As shown in Figure S8, the application of the precipitating TMB solution, even for a short time of 5 min (Figure S8b), resulted in significant alterations in the surface topography and coverage of the Ag/Si nanostructures with a layer of the TMB. After 30 min of incubation with the precipitating substrate, the coverage of the Ag/Si nanostructures was almost complete (Figure S8c) compared to the situation prior to the substrate application (Figure S8a). Thus, it seems that the layer of TMB inhibits the interaction of the incident light with the Ag/Si nanostructures underneath, reducing the Raman signal enhancement from them and eliminating the SERS phenomenon. It should be noticed that, when the complete survivin curve was run applying a 30 min incubation with the TMB substrate, the signals obtained were increasing inversely proportional to the survivin concentration; i.e., the signal decreased as the survivin concentration increased. Thus, to avoid such phenomena, a 5 min incubation of the Ag/Si nanostructured substrates with the TMB solution was adopted in the final protocol.

### 3.5. Analytical Characteristics of Survivin Assays

**Figure 6:**
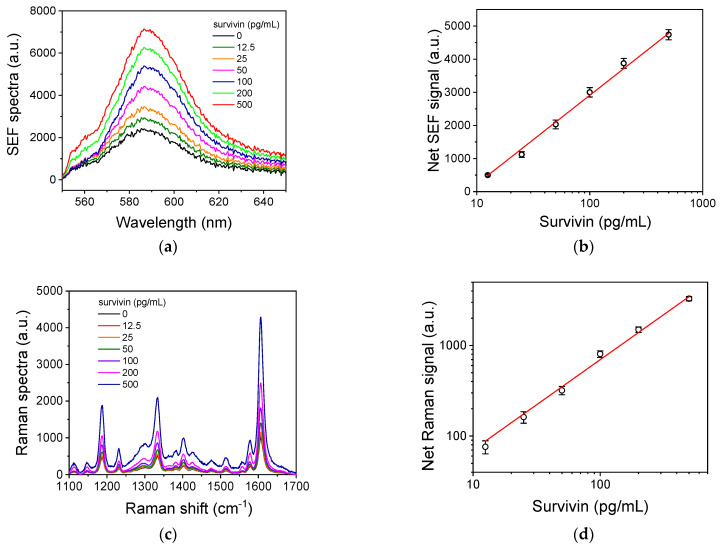
(**a**) SEF spectra received from Ag/Si substrates with dendrites for survivin calibrators from 0 to 500 pg/mL in assay buffer. (**b**) Survivin SEF calibration curve. (**c**) SERS spectra received from Ag/Si substrates with aggregates for survivin calibrators from 0 to 500 pg/mL in assay buffer. (**d**) Survivin SERS calibration curve. Each point is the mean value of 10 measurements from 3 replicate samples ± 3SD.

In Figure 6, characteristic SEF (Figure 6a) and SERS spectra (Figure 6c) corresponding to different survivin calibrators are presented, along with the respective calibration curves (Figure 6b and Figure 6d, respectively). The linear regression equation of the SEF curve was y = −2451(±26) + 2680(±21) × log[x], with a coefficient of regression of R^2^ = 0.998; and the linear regression equation of the SERS curve was log[y] = 0.85(±0.10) + 0.99(±0.04) × log[x], with a coefficient of regression of R^2^ = 0.993.

To determine the detection and quantification limits of each immunoassay approach, 10 replicate measurements of the zero calibrator were performed, and the signals corresponding to 5 and 10 standard deviations were correlated to the respective survivin concentrations using the SEF and SERS calibration curves. The detection limit for both detection approaches was 12.5 pg/mL, the quantification limit was 25 pg/mL, and the linear dynamic range was up to 500 pg/mL. It is important to note that the enzyme immunoassay developed using the same antibodies and conditions in microtiter plates had the same linear dynamic range and detection limit (Figure S9).

The effect of the serum in the assay performance was also determined. The presence of the serum resulted in a significant decrease in the SEF for all the calibrators, reaching in most cases a 50% signal reduction. On the other hand, the SERS signal for the calibrators in the human serum increased marginally (up to 10%) with respect to the values obtained for the calibrators in the buffer. This difference in the effect of the serum on the two detection methods is probably due to the different mechanisms of signal enhancement between them, and it might also reflect a different effect of the serum regarding substrates of different structures. However, neither the detection sensitivity nor the dynamic range of the assay were affected (Figure S10), enabling the detection of the survivin concentration on both healthy and cancer patients’ sera. The linear regression equation of the SEF curve in the serum was y = 1.83(±0.03) + 0.66(±0.01) × log[x], and the coefficient of regression was R^2^ = 0.998; that of the SERS curve in the serum was log[y] = 0.99(±0.12) + 0.94(±0.05) × log[x], and the coefficient of regression was R^2^ = 0.991. The reproducibility of the two immunoassay approaches was tested by preparing three samples corresponding to different concentration levels (20, 75, and 300 pg/mL) in spiked human serum. In particular, the intra-analytical repeatability was determined by analyzing the three samples in triplicate on the same day and calculating the coefficient of variation (CV), while the inter-analytical repeatability was determined by analyzing the same serum samples in duplicate in assays performed on four different days. The intra- and inter-assay CVs ranged from 5.2 to 8.9%, and from 6.7 to 9.6% for the SEF and SERS measurements, respectively. To determine the accuracy of the two detection approaches, recovery tests were performed by spiking the control samples with known quantities of survivin and determining their concentrations prior to and after the addition. The results presented in Table S2 and S3 for the SEF and SERS assays, respectively, reveal the accuracy of the determination with both detection approaches, with the percent recovery values ranging from 85.0 to 112%.

### 3.6. Comparison with Literature Methods

There are only a few reports in the literature for the determination of survivin based on immunosensors. For example, a single layer of Au–Ag nanoboxes that was created by self-assembly at an oil–water interface was transferred on a poly(dimethyloxy siloxane) substrate and used as solid-support of an antibody array for the simultaneous determination by SERS of survivin and the squamous cell carcinoma antigen (SCCA) [40]. For the detection, Au–Ag nanoshells were labeled with two different Raman reporters and then modified with the antibodies against the two markers to facilitate their simultaneous determination. The detection limits achieved were 5 and 6 pg/mL for the survivin and SCCA, respectively, and the linear dynamic range was up to 10 μg/mL. However, the assay took more than 4 h to complete and included two steps, one performed at 37 °C and the other at 4 °C. In another report, gold nanoparticles were functionalized with an anti-survivin antibody and used to detect survivin in human urine based on the shift in absorbance of the nanoparticles caused by aggregation upon the binding of the analyte [41]. The results were compared to an ELISA and, although the exact analytical characteristics of the two methods are not mentioned, effective discrimination between the samples from healthy individuals and those from bladder cancer patients is demonstrated by visual inspection of the gold nanoparticle/urine sample mixture after 15 min. Finally, a piezometric biosensor was employed for survivin detection in cell lysate solutions from a human astrocytoma (glioblastoma) U-87MG cell line culture medium by direct binding (20 min) to antibody-modified Au-covered quartz crystal piezoelectrodes [38,42]. A limit of detection of 1.7 nM was determined that corresponds to 28 pg/mL. As can be concluded by the data presented in Table 1, compared to the literature publications for survivin determination involving biosensors, the methods developed based on SEF or SERS have comparable detection limits with the literature methods, achieved following a much easier assay protocol as well as a less cumbersome and expensive substrate fabrication method.

## 4. Conclusions

A comparison of the nanostructured Ag/Si substrates with two different types of Ag nanostructures regarding the immunochemical determination of survivin via SEF and SERS has been presented. The assay employed a pair of specific antibodies, from which the detection one was biotinylated and was combined with streptavidin labeled with the fluorescent dye Rhodamine Red-X for the SEF measurements or with streptavidin labeled with peroxidase and a precipitating substrate (TMB) that provided strong Raman signals for the SERS measurements. The substrates with Ag dendrites provided higher SEF signals, while those substrates with Ag aggregates provided higher SERS signals and were therefore selected for the respective measurements. The detection limits achieved with both detection approaches were 12.5 pg/mL, with the linear dynamic range to extend from 25 to 500 pg/mL, covering the concentration ranges of both healthy and ovarian cancer patients. The substrates employed for the SEF measurements were prepared by a one-step procedure on silicon wafers, and the measurements were performed by a low-cost optical set-up for the excitation and collection of the emitted fluorescence signal. On the other hand, the preparation of the SERS substrates involved a three-step preparation procedure and a high-cost benchtop instrument for the acquisition of the signal. Since there was not any obvious advantage of the SERS compared to SEF measurements, the latter seem to be more suitable for application at the point of care or in low-resource environments, at least as far as the immunochemical detection methods are considered.

## Figures and Tables

**Table 1:** Comparison of the developed method with other literature methods.

Substrate	Detection Principle	LOD (pg/mL)	Dynamic Range	Analysis Time (min)	Ref.
Au–Ag nanoboxes	SERS	5.0	5 pg/mL–10 μg/mL	240	[40]
Au nanoparticles	LSPR	-	-	15	[41]
Au modified quartz crystal	piezoelectric	28	28–500 pg/mL	60	[42]
**SiNWs-dendrites/** **SiNWs-aggregates**	**SEF** **SERS**	**12.5**	**25–500 pg/mL**	**15** **0**	**This work**

## Data Availability

The data presented in this study are available upon request from the corresponding author. The data are not publicly available due to privacy issues.

## Supplementary Materials

The following supporting information can be downloaded at https://www.mdpi.com/article/10.3390/bios14100479/s1, Figure S1: Schematic of the steps followed for the fabrication of silicon nanowires decorated with (a) Ag dendrites or (b) Ag aggregates; Figure S2: Top view SEM image of 500 nm-long Si nanowires decorated with approximately 150 nm-long Ag aggregates; Figure S3. (a) Net SEF spectra received for a calibrator containing 100 pg/mL survivin from Ag/Si substrates with aggregates (red line) or from Ag/Si substrates with dendrites (blue line). (b) SERS spectra received for a calibrator containing 100 pg/mL survivin from Ag/Si substrates with aggregates (red line) or from Ag/Si substrates with dendrites (blue line); Figure S4: Normalized SERS for crystal violet (CV) with 514 nm laser on Ag aggregates (upper plot) and Si-reference (lower plot). For the case of Ag aggregate substrates, a series of 60 measurements with 1 sec acquisition time were taken; Figure S5: SEF signals obtained from nanostructured Ag/Si substrates with Ag dendrites for the zero calibrator (orange columns) and a calibrator containing 50 pg/mL (green columns) using the following buffers for the preparation of capture antibody solution: (1) phosphate buffer 50 mM, pH 7.4; (2) carbonate buffer 50 mM, pH 9.2; and (3) Tris-HCl buffer 10 mM, pH 8.25, 0.1 M NaCl. Each point is the mean of three samples ± SD; Figure S6: Survivin calibration curves obtained from nanostructured Ag/Si substrates with Ag dendrites using as assay buffer phosphate buffer 50 mM, pH 7.4 (triangles), or Tris-HCl buffer 50 mM, pH 8.25 (squares). Both buffers contained 10 g/L BSA, 9 g/L NaCl, 0.5 g/L NaN3. Each point is the mean of three samples ± SD; Figure S7: Raman spectra received from Ag/Si substrates with aggregates for survivin calibrators with concentration 0 (black line) and 50 pg/mL (red line), as well as the net signal for the 50 pg/mL calibrator (blue line) using as label 4-MBA-Au-streptavidin (a) or streptavidin labeled with peroxidase in combination with TMB precipitating substrate (b). Each point is the mean value of 5 measurements from 3 replicate samples ± 3SD; Figure S8: Characteristic top view SEM images received prior to (a) and after the application of TMB substrate for 5 (b) or 30 min (c) onto Ag/Si substrates modified with anti-survivin capture antibody, which were incubated with a 200 pg/mL survivin calibrator and a 25 ng/mL streptavidin-peroxidase solution; Figure S9: Survivin ELISA calibration curve. Each point is the mean of four replicates ± SD; Figure S10: (a) SEF calibration curve received with survivin calibrators in human serum. (b) SERS calibration curve received with survivin calibrators in human serum. Each point is the mean value of 5 measurements from 3 replicate samples ± 3SD; Table S1: Calculated SERS aEF values for the surface with the Ag aggregates for the several peaks observed in the CV spectrum; Table S2: Percent recovery values of survivin added to human serum determined by the SEF assay; Table S3: Percent recovery values of survivin added to human serum determined by the SERS assay.
