# Supplementary material for: Comparison of Survivin Determination by Surface-Enhanced Fluorescence and Raman Spectroscopy on Nanostructured Silver Substrates
Source: Biosensors (Basel). 2024 Oct 6;14(10):479. doi: 10.3390/bios14100479 (PMC11506520; doi:10.3390/bios14100479)
Supplement: Supplementary file 1 [file biosensors-14-00479-s001.zip › biosensors-3147850-supplementary.pdf]

biosensors

Supplementary material

Comparison of Survivin Determination by Surface-Enhanced Fluorescence and Raman Spectroscopy on Nanostructured Silver Substrates

Georgia Geka [1,2], Anastasia Kanioura [1,*], Ioannis Kochylas [3], Vlassis Likodimos [3], Spiros Gardelis [3], Anastasios Dimitriou [4], Nikolaos Papanikolaou [4], Anastasios Economou [2], Sotirios Kakabakos [1] and Panagiota Petrou [1,*]

[1] Immunoassays/Immunosensors Laboratory Institute of Nuclear & Radiological Sciences & Technology, Energy & Safety, NCSR "Demokritos", 15341 Aghia Paraskevi, Greece; g.gkeka@rrp.demokritos.gr (G.G.); skakab@rrp.demokritos.gr (S.K.)
[2] Department of Chemistry, National and Kapodistrian University of Athens, University Campus, 15771 Athens, Greece; aeconomo@chem.uoa.gr
[3] Section of Condensed Matter Physics, Department of Physics, National and Kapodistrian University of Athens, University Campus, 15784 Athens, Greece; ikochyla@phys.uoa.gr (I.K.); vlikodimos@phys.uoa.gr (V.L.); sgardelis@phys.uoa.gr (S.G.)
[4] Institute of Nanoscience & Nanotechnology, NCSR "Demokritos", 15341 Aghia Paraskevi, Greece; a.dimitriou@inn.demokritos.gr (A.D.); n.papanikolaou@inn.demokritos.gr (N.P.)
* Correspondence: nkanioura@ipta.demokritos.gr (A.K.); ypetrou@rrp.demokritos.gr (P.P.); Tel.: +30-210-650-3819 (P.P.)

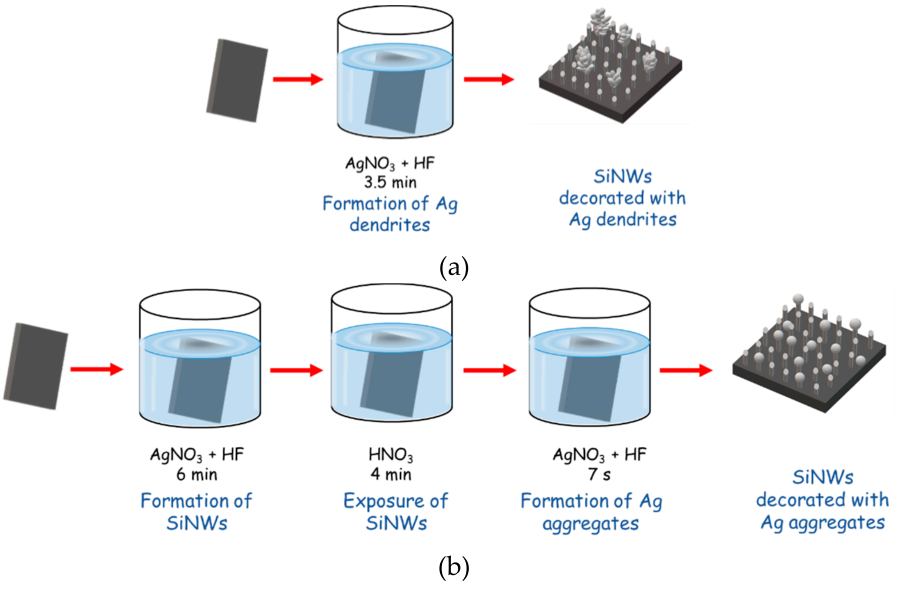

Figure S1. Schematic of the steps followed for the fabrication of silicon nanowires decorated with (a) Ag dendrites or (b) Ag aggregates.

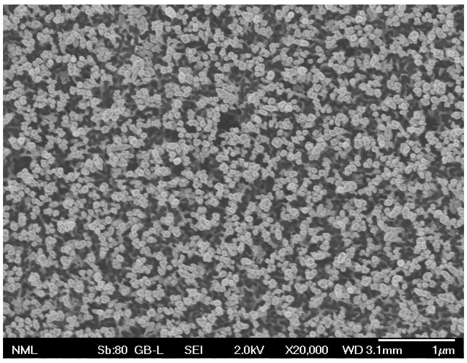

Figure S2. Top view SEM image of 500-nm long Si nanowires decorated with approximately 150-nm long Ag aggregates.

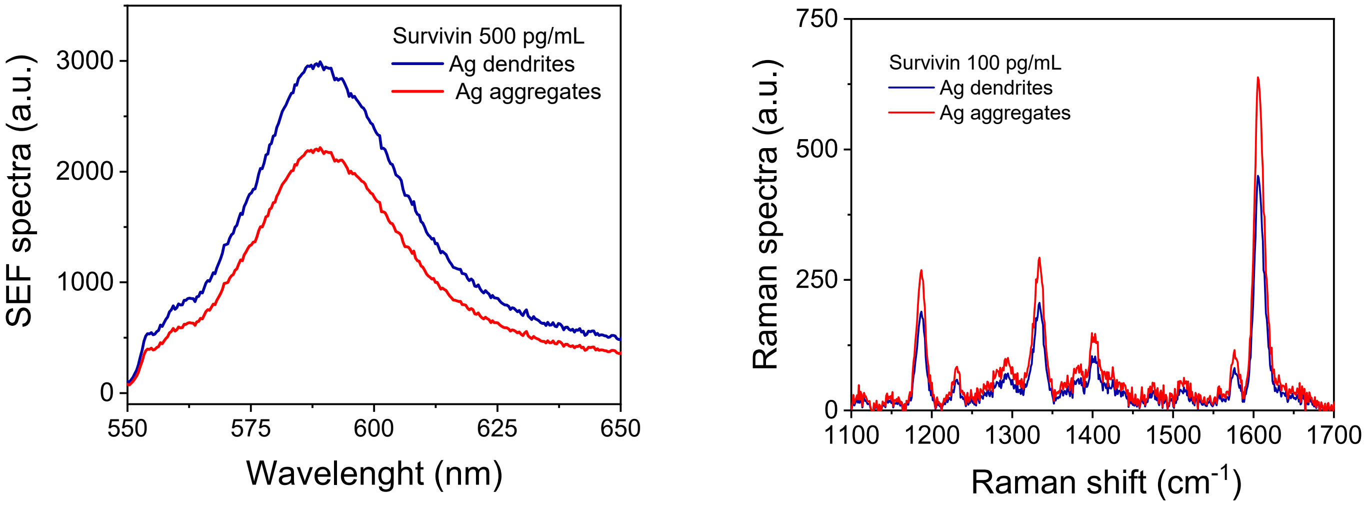

(a) (b)

Figure S3. (a) Net SEF spectra received for a calibrator containing 100 pg/mL survivin from Ag/Si substrates with aggregates (red line) or from Ag/Si substrates with dendrites (blue line). (b) SERS spectra received for a calibrator containing 100 pg/mL survivin from Ag/Si substrates with aggregates (red line) or from Ag/Si substrates with dendrites (blue line).

Calculation of SERS enhancement factor (EF)

For the calculation of the SERS Enhancement Factor (EF) we used the method proposed in ref. 34. This method is based on the following formula:

$$aEF = \frac{I_{SERS}/(P_{SERS} \times T_{SERS} \times N_{SERS})}{\frac{I_{RS}}{(P_{RS} \times T_{RS} \times N_{RS})}}$$

where I_{SERS} expresses analyte Raman scattering intensity on SERS-active substrates (Ag aggregates in our case) and I_{RS} expresses the Raman intensity from the analyte deposited on a non-enhancing surface (plain Si wafer). P_{SERS} and P_{RS} represent the power of an excitation laser, which in our case is the same for SERS-active and non-active substrates, T_{SERS} and T_{RS} are the acquisition times for each case respectively. N_{SERS} and N_{RS} are the number of molecules illuminated by the incident laser spot sampled on the SERS-active substrate and the non-enhancing substrate, respectively. We used the same optical parameters (the wavelength of 514 nm, the 20× objective lens, spectrometer) to acquire all spectra. In order

to detect Raman signal at the non-enhancing surface we used a significantly higher analyte solution. The measured SERS spectra after smoothing and baseline correction normalized according to the formula above are displayed in Figure S3.

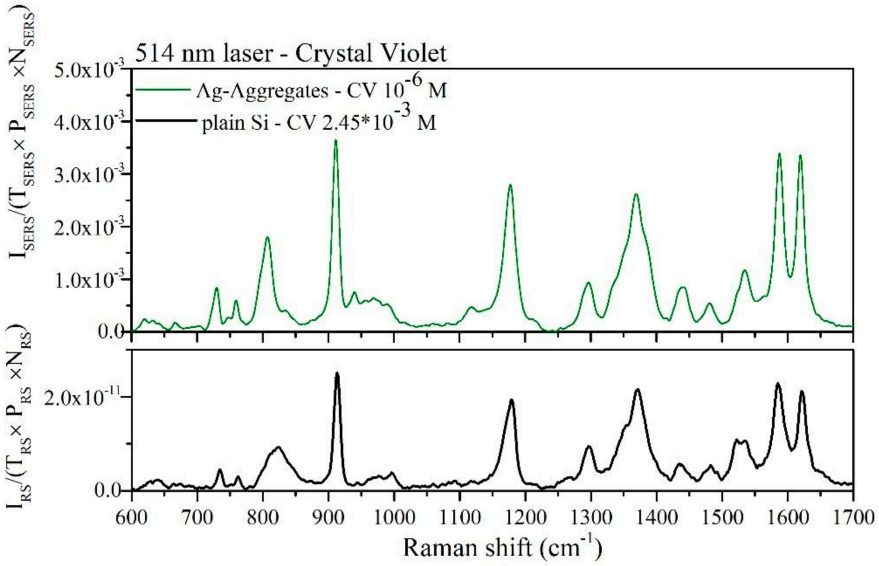

Figure S4. Normalized SERS for crystal violet (CV) with 514 nm laser on Ag aggregates (upper plot) and Si-reference (lower plot). For the case of Ag aggregates substrates, a series of 60 measurements with 1 sec acquisition time were taken.

Table S1 shows the results for the aEFs for some of the intense crystal violet (CV) Raman bands displayed in Figure 1 for Ag aggregates. The highest aEF, 1.8×10^8, of the Ag aggregates was found at 1586.78 cm^{-1}.

Table S1. Calculated SERS aEF values for the surface with the Ag aggregates for the several peaks observed in the CV spectrum.

**Peak wavenumber (cm^{-1})**	**Calculated aEF value ($\times 10^8$)**
914.16	1.4 (±0.1)
1174.51	1.5 (±0.2)
1370.89	1.2 (±0.1)
1586.78	1.8 (±0.2)
1619.19	1.6 (±0.2)

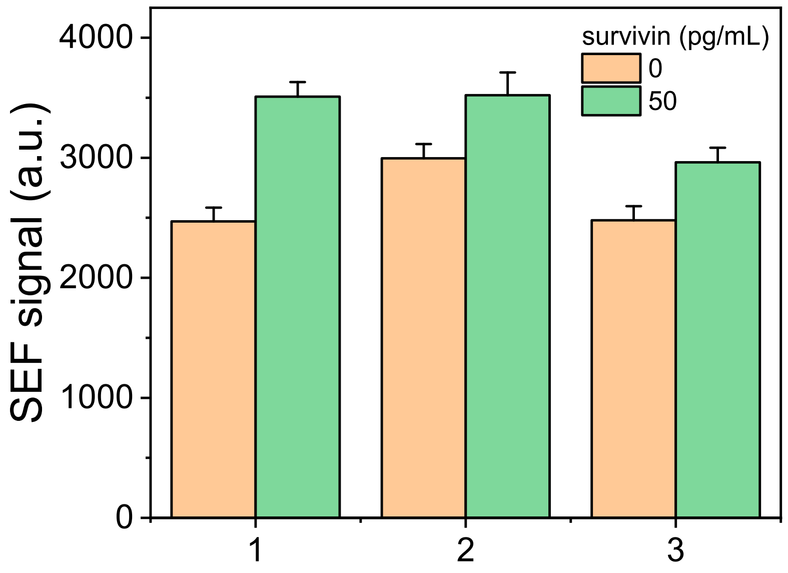

Figure S5. SEF signals obtained from nanostructured Ag/Si substrates with Ag dendrites for the zero calibrator (orange columns) and a calibrator containing 50 pg/mL (green columns) using the following buffers for the preparation of capture antibody solution: (1) phosphate buffer 50 mM, pH 7.4; (2) carbonate buffer 50 mM, pH 9.2; and (3) Tris-HCl buffer 10 mM, pH 8.25, 0.1 M NaCl Each point is the mean of three samples ± SD.

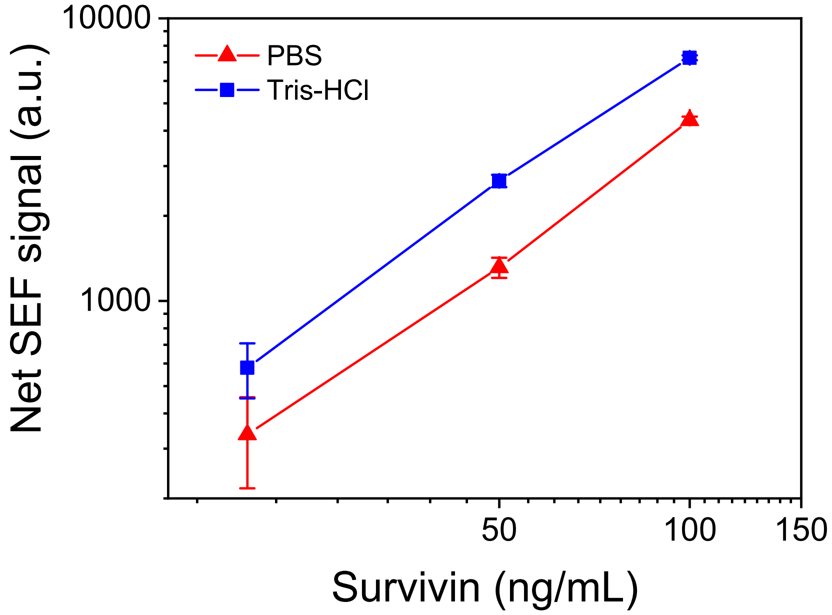

Figure S6. Survivin calibration curves obtained from nanostructured Ag/Si substrates with Ag dendrites using as assay buffer phosphate buffer 50 mM, pH 7.4 (triangles) or Tris-HCl buffer 50 mM, pH 8.25 (squares). Both buffers contained 10 g/L BSA, 9 g/L NaCl, 0.5 g/L NaN3. Each point is the mean of three samples ± SD.

Figure S7. Raman spectra received from Ag/Si substrates with aggregates for survivin calibrators with concentration 0 (black line) and 50 pg/mL (red line), as well as the net signal for the 50 pg/mL calibrator (blue line) using as label 4-MBA-Au-streptavidin (a) or streptavidin labelled with peroxidase in combination with TMB precipitating substrate (b). Each point is the mean value of 5 measurements from 3 replicate samples ± 3SD.

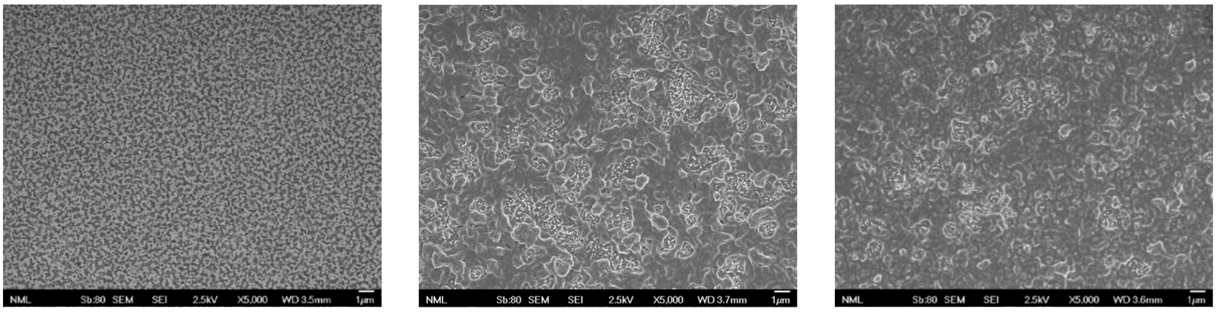

(a) (b) (c)

Figure S8. Characteristic top view SEM images received prior to (a) and after the application of TMB substrate for 5 (b) or 30 min (c) onto Ag/Si substrates modified with anti-survivin capture antibody which were incubated with a 200 pg/mL survivin calibrator and a 25 ng/mL streptavidin-peroxidase solution.

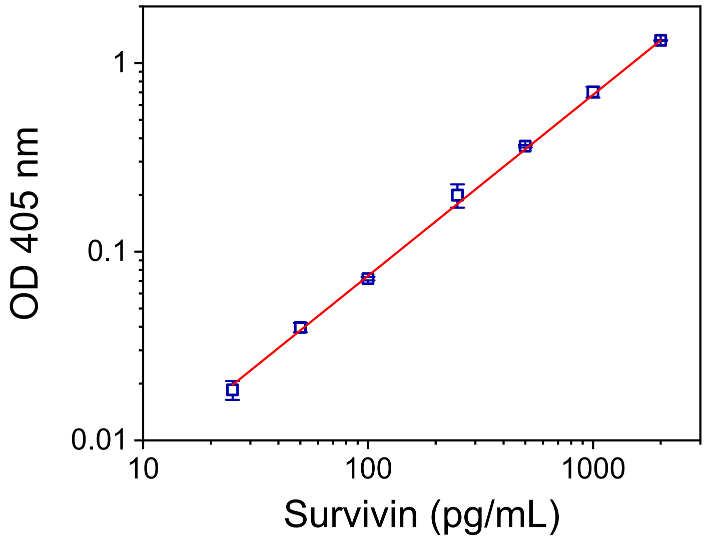

Figure S9. Survivin ELISA calibration curve. Each point is the mean of four replicates ± S

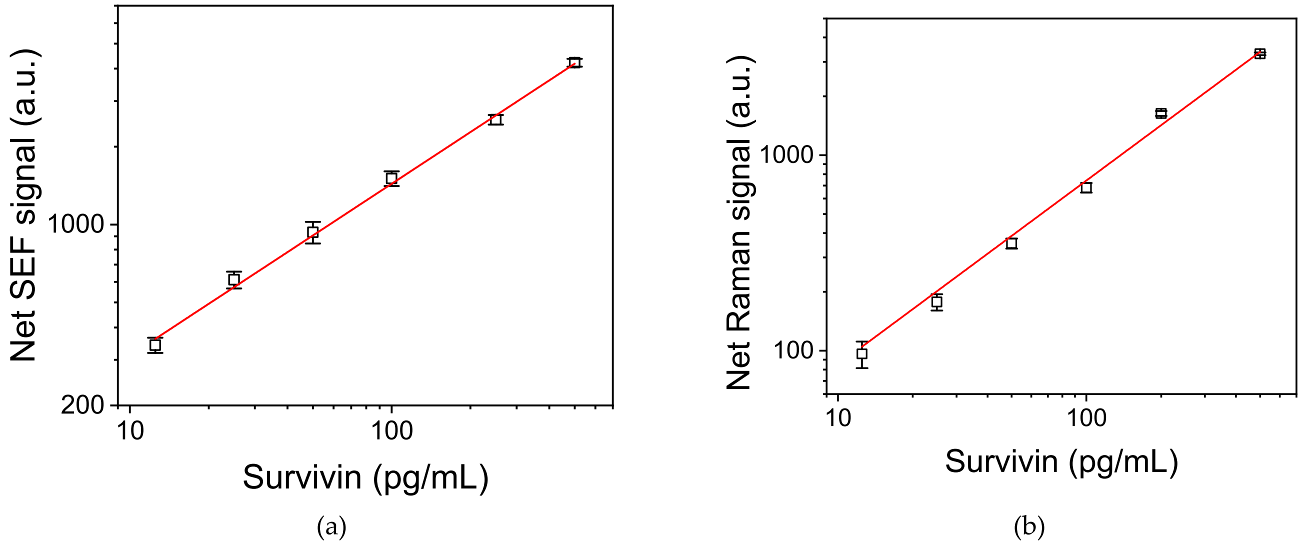

Figure S10. (a) SEF calibration curve received with survivin calibrators in human serum. (b) SERS calibration curve received with survivin calibrators in human serum. Each point is the mean value of 5 measurements from 3 replicate samples ± 3SD.

Table S2. Percent recovery values of survivin added to human serum determined by the SEF assay.

Initial concentration (pg/mL)	Concentration added (pg/mL)	Concentration determined (pg/mL)	%Recovery
	10	9.5 ± 0.6	95.0 ± 6.0
20	20	22.5 ± 1.0	112 ± 5.0
	40	37.0 ± 2.0	92.5 ± 5.0
	30	32.0 ± 2.0	107 ± 6.7
75	75	73.0 ± 2.0	97.3 ± 2.6
	150	160 ± 3.0	107 ± 1.9

Table S3. Percent recovery values of survivin added to human serum determined by the SERS assay.

Initial concentration (pg/mL)	Concentration added (pg/mL)	Concentration determined (pg/mL)	%Recovery
	10	12.0 ± 0.5	120 ± 5.0
20	20	17.0 ± 1.0	85.0 ± 5.0
	40	43.0 ± 1.0	107 ± 2.5
	30	28.0 ± 2.0	93.3 ± 6.7
75	75	78.0 ± 1.0	104 ± 1.3
	150	135 ± 5.0	90.0 ± 3.3
